# Exploring digital health technology in rheumatology: a scoping review on benefits, challenges, and future possibilities

**DOI:** 10.1016/j.ero.2026.01.001

**Published:** 2026-01-29

**Authors:** Esther Ramlakhan, Eiman Soliman

**Affiliations:** 1 Department of Internal Medicine, Rheumatology Unit, San Fernando General Hospital, San Fernando, Trinidad and Tobago; 2 Rheumatology and Clinical Immunology, Department of Internal Medicine, Faculty of Medicine, Alexandria University, Alexandria, Egypt

## Abstract

**Objective:**

The objective of this study is to identify the benefits associated with the integration of digital health technology within rheumatology care, the challenges associated with its implementation, and its future potential to transform rheumatology practice in an evolving global society.

**Methods:**

A scoping review was conducted utilising the PRISMA (Preferred Reporting Items for Systematic reviews and Meta-Analyses) extension for Scoping Review guidelines. The Cochrane, Medline, Embase, and Wiley Online Library databases were searched to identify studies on digital health technology in rheumatology practice within the last 10 years (2015-2025). Study outcomes were summarised using a narrative, descriptive analysis.

**Results:**

Forty-six articles were included in the final review. The main forms of digital health technology included telehealth/telemedicine, electronic health records, and electronic patient-reported outcome measures, digital health intervention applications, self-assessment and monitoring applications, digital diagnostic decision support systems, and cloud platforms. Benefits included improved management of chronic diseases with increased flexibility, time saving, and high levels of patient satisfaction. Challenges identified included difficulty with usage due to lack of digital literacy and technology malfunction, disparities with patient access to technology, physician burnout with additional monitoring requirements, increased training, hardware and software requirements, and privacy and security concerns. Projected opportunities included the generation of a big data scenario with increased linkages, allowing opportunities for evidence-based research and data collection in real time, the identification of patient subgroups that may benefit from more frequent care and improved patient empowerment and communication.

**Conclusions:**

Digital health technology has potential value with benefits to self-management, clinical care and research opportunities. This can lead to health advancements at an individual and population level for both rheumatology practice and general healthcare once issues identified arising from its implementation have been adequately addressed.

## INTRODUCTION

Digital technology has led to the rapid advancement of the health industry in the 21st century by improving patient outcomes, enhancing access to care, and streamlining clinical workflows [1]. Following the aftermath of the COVID-19 pandemic, which resulted in increasing health care costs and sustained societal isolation, etc, the need for remote and digital solutions in healthcare has become increasingly apparent, with rheumatology becoming a key area involved in this digital transformation [2]. The increased availability of various forms of digital technology in the 21st century allows for improvements in healthcare delivery to be made possible. There is currently a lack of consensus regarding the definition of digital health and digital health technology, with several sources noting varying definitions [3,4]. According to the FDA 2020, digital health includes categories such as ‘mobile health (mHealth), health information technology (IT), wearable devices, telehealth/telemedicine, and personalized medicine [5].’ A mapping review of definitions surrounding digital health concluded that digital health focuses mainly on the proper use of technology for improving patient health and wellbeing rather than focusing on the type of technology itself [6]. The term ‘Ehealth’, however, can be described as ‘an umbrella term including mHealth, remote health (telehealth), and ubiquitous health (uHealth) [3].’ In Rheumatology practice, several forms of digital health technologies have emerged, eg, electronic health records (EHRs), telemedicine, innovative mobile applications, and artificial intelligence (AI), to name a few [7]. The major advantage of such technology is the enabling of seamless collaboration between patients and providers in the management of chronic rheumatic conditions. Drawbacks, however, include the potential to exacerbate disparities in access to care if not implemented thoughtfully, the need to address concerns around data privacy and security, and the challenge of ensuring these technologies are user-friendly and integrated seamlessly into clinical workflows [7]. Major projected benefits associated with the implementation of digital health technology in rheumatology care include the ability to collect high-resolution longitudinal data, the facilitation of remote monitoring and patient engagement, and the provision of personalised interventions adjusted to individual requirements and preferences [6]. The ability to collect data in real time opens the door for evidence-based research and the generation of a big data scenario with increased intersectoral connectivity, ultimately leading to rapid breakthroughs in medicine, diagnostics and overall patient management [8]. Digital health interventions (DHIs) in rheumatology hold immense promise for transforming the landscape of care, offering innovative solutions to address the challenges of managing chronic rheumatic diseases and improving patient outcomes [9].

There is a need for further studies on the topic of digital health technology in the form of systematic reviews, real-world evidence from large-scale observational studies, and well-designed clinical trials to fully elucidate its role and impact in rheumatology practice [10]. It is a growing topic of interest amongst a wide range of healthcare professionals, researchers, and policymakers*.* This scoping review aims to provide an overview of the current state of available evidence on digital health technology in rheumatology, exploring its applications, benefits, challenges, and future potential. Overall, the main questions the review sought to answer included:

(1) What are the main forms of Digital Health Technology employed in rheumatology practice, and how they may impact patient care?
(2) What are the challenges arising out of the transition to digital care within rheumatology practice for both patients and physicians inclusive of barriers to implementation?
(3) What are future potential applications of Digital Health Technology in rheumatology?

## METHODS

The research questions for this review were derived from an initial search of the literature surrounding the subject area, which helped to determine the suitability of conducting a scoping review on the chosen topic. Study aims were completed by utilising university library resources to aid in searching relevant databases, searching grey literature and unpublished studies to find available evidence on the topic, and conducting a narrative analysis of the synthesised evidence. It was done using a systematic approach, reported with guidance from the PRISMA extension for scoping review guidelines [11]. The PRISMA Extension guidelines for scoping reviews eliminate the need for assessing risk of bias across studies. The review consisted of 2 independent reviewers who collaborated during the search and discussed the results. Standardised data extraction and storage tools Zotero and Covidence were used to assist in accessing, extracting, and organising data from the review. One reviewer mapped the results that were applied verbatim for the derivation of the outcomes and challenges, whereas results for the future implication section were derived both by verbatim and inferential interpretation of the data from the individual articles. Any discrepancies in interpretation were discussed between the reviewers.

### Study search and selection

A search was conducted utilising the Cochrane, Pubmed/Medline, Embase, and Wiley Online Library electronic databases during March to May 2025. A manual search through the relevant review articles was also conducted. The search terms inclusive of MeSH terms and their Boolean operators included ‘DIGITAL HEALTH AND RHEUMATOLOGY’, ‘TECHNOLOGY AND RHEUMATOLOGY’, and ‘’DIGITAL TECHNOLOGY AND RHEUMATOLOGY’. Additional search terms included ‘E-HEALTH AND RHEUMATOLOGY’. Study titles were examined for relevance and progressed to abstract screening, where they were screened according to the inclusion/exclusion criteria.

### Eligibility criteria

Studies meeting eligibility criteria included:

• Studies exploring the role of digital health technology in both paediatric and adult rheumatology practice,
• Studies conducted within the last 10 years (for relevance)
• English studies/studies translated into English language

Exclusion criteria consisted of:

• Expert opinion, editorials, and blogs
• Non-English studies
• Studies involving noninflammatory/nonautoimmune conditions mainly managed by primary care, eg, osteoarthritis
• Pilot studies and incomplete clinical trials

**Figure 1:**
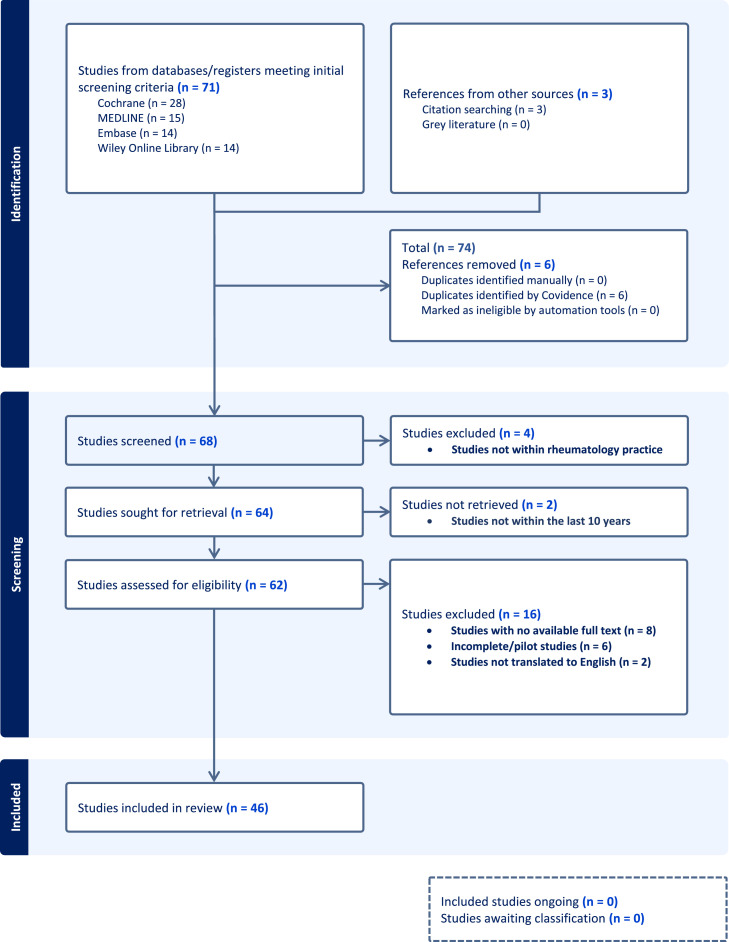
PRISMA (Preferred Reporting Items for Systematic Reviews and Meta-Analyses) flow diagram for studies included in the review.

Full-text articles were further screened for eligibility. A total of 74 articles met the initial screening criteria out of the 2150 references obtained. Forty-six (n = 46) studies were included in the final review. The rationale for studies included/excluded in the final review is reflected via the PRISMA (Preferred Reporting Items for Systematic reviews and Meta-Analyses) Flow diagram (Fig 1). The categories of digital health technology were derived mainly from assessing the aim and function of the technology as utilised in the individual articles. For example, studies exploring telemedicine/telehealth were grouped in 1 category, studies assessing the intervention of a digital application on a specific outcome, such as adherence, were grouped under DHI applications, whereas studies primarily aimed at assessing aspects of remote monitoring and self-management devices were grouped under self-assessment and monitoring apps. Studies grouped under the EHR/ePRO category were those whose primary aim was to assess the function of some aspect of an EHR/ePRO specifically. The studies exploring digital diagnostic decision support systems (DDSSs) and a cloud platform were placed in separate categories based on the function and nature of the technology being assessed, which did not fit entirely into the previous categories.

## RESULTS

### Summary of review characteristics

**Figure 2:**
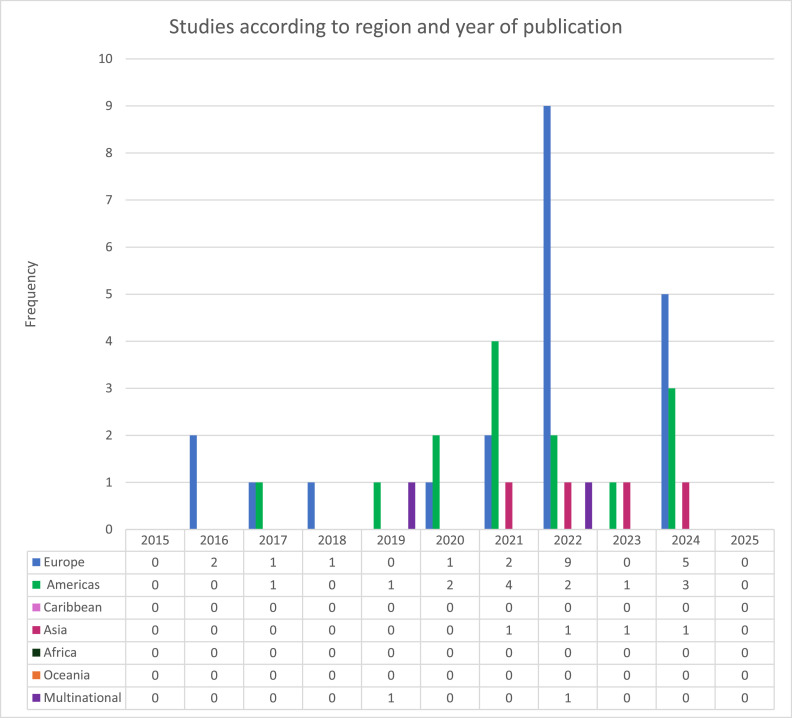
Primary studies according to geographical locations and year of publication.

**Figure 3:**
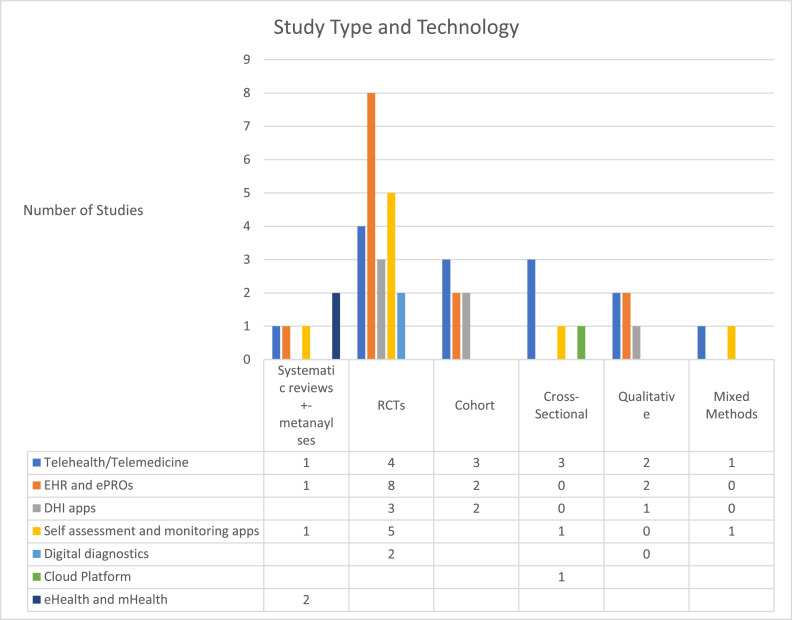
Characteristics of study type and technology revealed in the review. DHI, digital health intervention; EHR, electronic health record; ePROs, electronic patient-reported outcomes; RCT, randomised controlled trial.

This review yielded 46 (n = 46) articles of interest from 2015 to 2025 from various geographical locations (Fig 2). Five articles consisted of **secondary** sources of information in the form of systematic, scoping, and literature reviews. Forty-one (n = 41) articles consisted of **primary** studies, where 22 (n = 22) were randomised controlled trials (RCTs), 7 (n = 7) were cohort studies, 5 (n = 5) cross-sectional studies, 5 (n = 5) qualitative, and 2 (n = 2) mixed methods studies. A total (n = 13) or 31.7% of **primary** studies in the review focused on telehealth and telemedicine, (n = 12) or 29.3% of primary studies covered EHRs and electronic patient-reported outcomes (ePROs), (n = 6) or 14.6% focused on DHI apps, (n = 7) or 17.1% covered self-assessment and monitoring apps, (n = 2), or 4.9% of primary studies involved digital diagnostics, and (n = 1) or 2.4% involved a cloud platform (Fig 3).

### Summary of patient characteristics

**Figure 4:**
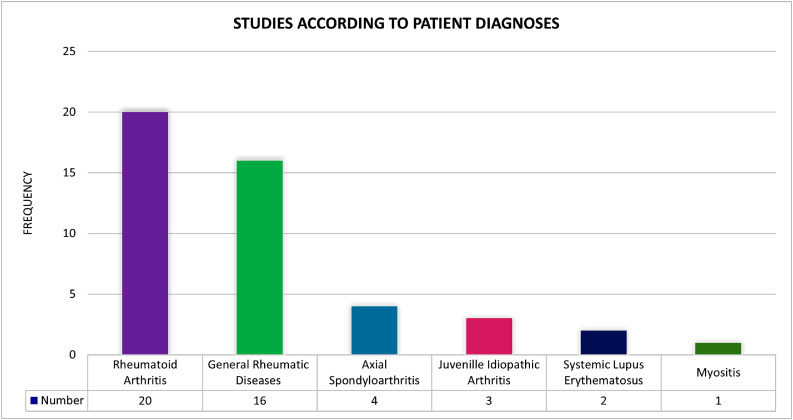
Studies according to patient diagnoses.

Of the 46 articles in the review, most studies involved patients with adult inflammatory arthritis (n = 28; 61.0%), where most studies focused specifically on patients with rheumatoid arthritis (RA). The remaining studies covered patients with systemic lupus erythematosus, myositis, juvenile idiopathic arthritis (JIA), and rheumatological diseases in general (Fig 4).

### Review of previous evidence synthesis

The secondary studies obtained from the literature search consisted of 2 systematic reviews, 2 systematic reviews with metanalyses, and 1 scoping review across the period 2017-2022 [8,[12], [13], [14], [15]]. Three studies focused on digital health technology in patients with chronic inflammatory arthritis [8,14,15], whereas 2 studies covered patients with chronic rheumatological conditions in general [12,13]. Studies focused on telehealth and telemedicine, EHRs or ePROs, remote monitoring interventions, and eHealth and mHealth in general. A summary of the previously synthesised evidence can be seen in Table 1.

**Table 1:** Previous evidence synthesis on digital health technology in rheumatology

Study ID	Design	Technology	Main findings
McDougall et al (2017) [12]	Systematic review (20 articles from 1995-2015)	Telerheumatology	Telerheumatology was found to be generally effective for the management of inflammatory and autoimmune diseases
			Evidence to support the effectiveness of Telerheumatology is, however, limited.
			Most conditions evaluated consisted of chronic arthritis, especially rheumatoid arthritis with less patients evaluated with connective tissue diseases or alternative diagnoses.
			Concerns raised included the effectiveness of Telerheumatology for initial diagnosis, concluding that Telerheumatology may be more suitable for follow-up of already established disease.
			Additional large-scale studies are needed with various forms of telemedicine in differing settings for generalisability. Further studies should also include a cost-effectiveness analysis and to determine the best uses of telemedicine for the diagnosis and management of these conditions.
Shelton et al (2021) [13]	Systematic review (70 articles from 2007-2020)	Electronic patient-reported outcomes (ePROs), and mHealth	The use of ePROs in the management of rheumatological conditions is a growing area of interest and has significant utility in clinical practice
			The most current condition evaluated included inflammatory arthritis.
			Generic ePROs were found to be utilised more frequently than disease-specific ePROs suggesting stability over the years.
			The use of ePROs have been found to improve patient outcomes and to correlate with physician reported disease activity.
			Further studies are recommended to assess the ideal frequency of ePRO collection, its potential to increase patient engagement, and improve patient outcomes, including disease activity.
Butler et al (2021) [14]	Systematic review and metanalysis (15 articles between 2008 and 2021)	eHealth and mHealth interventions in Juvenile Idiopathic Arthritis	50% of studies included symptom monitoring via obtaining real-time data using health applications, web-based portals or electronic diaries to monitor pain or health-related quality of life (HRQoL).
			20% of interventions pointed towards physical activity promotion via using a web-based programme or a wearable activity tracker where the web-based programme demonstrated increased endurance time, physical activity levels, and moderate to vigorous physical activity.
			The final 30% of interventions pointed towards the enhancement of self-management via web-based programmes, or apps, facilitating a minor effect, reducing pain severity and increasing disease awareness and self-efficacy. These results, however, were not statistically significant.
			Further research is required on the long-term effects of real-time monitoring, associated web-based interactions, a comparison amongst various self-management programmes, and the application of wearable technologies as an objective measurement for monitoring physical activity before any recommendations can be given.
Bragazzi et al (2022) [8]	Systematic review (14 articles with undefined period)	Big data, artificial intelligence, digital and smart technologies in psoriatic arthritis	Big data analytical techniques can be used to analyse multidimensional datasets involving clinically heterogeneous and complex diseases opening the way for globally significant research.
			Digital and smart technologies and artificial intelligence can be harnessed for early interception, treatment, and management of chronic rheumatological diseases.
			Further recommendations for future research include a standardised methodology for assessing the impact of such technology on multiple diseases and addressing ethical issues that may arise including privacy concerns.
Doumen et al (2022) [15]	Systematic review and meta-analysis (45 articles between 2008 and 2022)	eHealth tools for remote monitoring in chronic arthritis	The review found generally high-reported engagement rates with eHealth tools for remotely monitoring disease activity and impact in patients with chronic arthritis.
			Engagement with eHealth tools was found to decline over time to a highly variable degree and data came mostly from strictly controlled research settings which possibly underestimated the issue of attrition.
			Future studies are recommended with standardised measures of engagement, with preference for assessment in a clinical practice setting.

### Summary of studies according to technology

The main forms of digital health technology as revealed by this review included: telemedicine and telehealth, EHRs, and ePROs, DHI applications, self-assessment and monitoring applications, digital diagnostics, and cloud platform technology.

### Telemedicine and telehealth

Thirteen studies from the review covered telehealth/telemedicine. Most studies were noted to evaluate the role of telemedicine in improving patient outcomes, including disease activity. Two studies noted improvement in patient outcomes/disease activity with telemedicine [16,17], whereas 2 noted no significant differences compared with standard care [18,20]. One cross-sectional study utilising univariate and multivariate analysis also did not show any significant differences in outcomes and quality measures with telemedicine in comparison to standard care [19]. One study noted telemedicine care to be worse than usual care as rated by both patients and physicians [21] largely due to reasons such as lack of building a trusting relationship, feeling ‘shoved off’ from having a physical consultation, concerns for inaccurate assessments via phone, and the possibility of technical difficulties occurring during the teleconsultation. Additional studies evaluated the perceptions of patients and physicians with telemedicine/telehealth services, in which the majority revealed high levels of patient satisfaction [17,18,20,22], whereas the remaining studies focused on the impact and applications of telemedicine in the face of the COVID-19 pandemic and in the postpandemic era [[23], [24], [25]]. Recurrent themes regarding the challenges faced by the implementation of telemedicine into patient care included the existence of technical difficulties, additional training and support required for use [17,18,25,26], disparities caused by decreased access to care and digital literacy level [16,[21], [22], [23],^27^,^28^], the unsuitability of telemedicine for managing disease flares and new patient misdiagnoses [20,21,26], health care professional burn out with extra duties [21,22], data security concerns [21,25], and the unwillingness to utilise telemedicine and telehealth services for the elderly and those unfamiliar with the technology [16,22,28]. Future implications concerning telemedicine and telehealth generally reflect positively on the incorporation of telehealth and telemedicine into standard care, however, with large efforts recommended into shared decision making with patients and physicians [18,21,26], bridging the literacy gap for patients with training and support tools [21,23,28], increasing access to services available and establishing adequate criteria for incorporating telemedicine and telehealth services largely into outpatient care [16,21,24,25,28]. A summary of the studies covering telehealth and telemedicine can be seen in Supplementary Table S1, Appendix A.

The studies covering telehealth and telemedicine consisted of 4 RCTs [[16], [17], [18],^20^], 3 cohort studies [23,24,27], 3 cross-sectional studies [19,25,28], 2 qualitative studies [22,26], and 1 mixed methods study [21]. This presents a mixture of level I-IV evidence, with the majority consisting of level I-III evidence.

### EHRs and ePROs

Twelve studies from the review covered EHRs/ePROs. Most studies assessed the use of ePROs alongside mobile applications in patient monitoring, their impact on potentially improving disease activity and perceptions of the use of ePROs from either a patient or physician perspective. Two studies did not show any significant improvements associated with the use of mobile applications with ePRO data to improve disease activity [29,30], whereas 1 study showed only short-term improvement [31]. Patients’ and physician’s assessment of ePRO/EHR services were generally positive, with high compliance rates to electronic recording amongst patients [30,[32], [33], [34], [35], [36]]. Physicians also expressed that ePROs contributed to time saving. Additional studies investigated the reliability of ePRO/EHR data relating both accuracy and equivalence to conventional approaches [37,38]. Challenges revealed from ePRO/EHR use included a lack of consensus regarding ideal ePRO collection [13,32], difficulties arising from a lack of digital literacy from both patients and physicians, lack of medical literacy on the part of programmers and IT experts [30], [35], [39], [40], technical difficulties during usage of applications to gather ePROs, and the lack of compensation of medical staff for the extra work needed to interact with these platforms utilising EHR/ePRO data [39,40]. The projected use of extensive resources needed for implementation of these systems, eg, programmers, staff training, hardware, and software, was also stated as a major setback for administrators and stakeholders [29], [35]. Another major issue is the lack of standardisation of global medical data, eg, evolving International Classification of Diseases codes and varying definitions of disease, which contributes to differences in EHR/ePRO coding, which may only be specific to the centre/site of implementation [37]. Despite these challenges, future possibilities revealed by the review focused mainly on the ability to collect dense, real-time data on patients, which has many benefits. These benefits include: EHR/ePROs data potentially serving as a performance index [29], creating allowances for adverse event/patient improvement data to be made readily available, opening opportunities for evidence-based research and data collection [31,32,37], and the identification of patient subgroups which may benefit from physical routine care vs remote care [34]. A summary of the studies involving EHR/ePRO data can be seen in Supplementary Table S2, Appendix A.

Studies from the review covering EHR/ePROs comprised of 8 RCTs [[29], [30], [31],^32^,^33^,[38], [39], [40]], 2 cohort studies [37] where 1 utilised RCT data [34], and 2 largely qualitative studies [35,36]. This consists of level I-IV evidence, with the majority consisting of level I-II evidence.

### DHI applications

Of the 6 studies that mainly focused on digital intervention applications, most studies assessed the impact of the DHI applications on the disease in question in terms of efficacy, safety, and feasibility. Three studies revealed varying levels of improvement with DHIs on disease activity [[41], [42], [43]], whereas 1 study failed to show an improvement with DHI app use in increasing patient adherence with Disease Modifying Anti-Rheumatic Drugs to improve disease activity [44]. Studies which showed improvement in disease activity also showed high levels of patient adherence to DHI use with resultant increased acceptability and efficacy [[41], [42], [43]]. Studies also investigated patient acceptance for DHI use, revealing communication with health care professionals to be a very important feature [[43], [44], [45], [46]]. Challenges encountered via the implementation of DHIs included patient reluctance to transition from traditional to digital care [41] and physician scepticism regarding the inability to replicate aspects of in-person care, eg, concerns regarding suboptimal care due to lack of physical monitoring [46]. The increased need for patient monitoring to ensure correct app use without physician compensation was also a challenge [45]. Additional challenges included a lack of digital literacy leading to nonuse, app malfunction, and lack of resources to facilitate app use, eg, storage space [41,42]. There is also a lack of studies regarding DHIs to justify the projected benefits of app use [14,45]. Most studies agree that patient empowerment is needed to increase utilisation of digital intervention apps [42,45,46]. Workshops for both patient and physician with training to use these apps are highly recommended [42]. Studies also cited physician involvement as crucial to patient motivation for the use of DHI applications and recommend automated messages to increase patient utilisation without direct healthcare provider involvement [43,45,46]. Personalisation of applications was also recommended [46].

Of the studies in the review investigating DHI applications, 3 comprised of RCTs [[42], [43], [44]], 2 were cohort studies [41,45], and 1 consisted of a qualitative study [46]. Most studies investigating DHIs consisted of either Level I or II evidence, which represents high-level evidence. A summary of the studies investigating DHIs can be seen in Supplementary Table S3, Appendix A.

### Self-monitoring and assessment tools

Most studies investigating self-assessment and monitoring tools focused on the efficacy of such in patient management, whereas 1 study investigated access and uses of eHealth in general [47]. Five studies reported improved patient outcomes with the use of self-assessment and monitoring applications, related to high levels of patient satisfaction [[48], [49], [50], [51], [52]]. Challenges encountered included nonuse due to a lack of digital health literacy and, additionally, due to perceived symptom control, where patients who were in a state of remission did not use the self-assessment/monitoring apps [48]. This also affected patient adherence, which was reported as nonoptimal in few studies with high rates of loss to follow-up [[47], [48], [49], [50],^53^]. In 1 study, patients felt the application prompts were an unwelcome reminder of their diseased state [52]. An additional reason for nonuse included concerns regarding data privacy and security [47]. Physicians were also sceptical regarding the use of self-monitoring/assessment apps, where they mostly feared an increased workload, opining that they lost control over patients with the introduction of these apps, and they were also resistant to change [47,53]. Application malfunction was also identified as a challenge [51]. Recommendations to improve future practice included the integration of these applications into EHRs/ePROs [53], further studies to determine the cost effectiveness of such applications given the high levels of nonuse and the incorporation of patient and physician input into the development of these applications.

The studies investigating self-monitoring and assessment tools comprised of 5 RCTs [[48], [49], [50], [51], [52]], 1 cross-sectional study [47], and 1 mixed methods study [53]. This represents mainly level I-II studies, with a mixture of 1 level III and 1 level IV study, which can be described as relatively strong evidence. A summary of studies describing self-assessment and monitoring apps can be seen in Supplementary Table S4, Appendix A.

### Digital DDSSs

These consisted of 2 cross-over RCTs investigating a DDSS: a mobile AI-based symptom checker (Ada) and a web-based self-referral tool (Rheport). The first study investigated the potential impact of the DDSS alongside patient experiences and acceptability, whereas the second study attempted to evaluate the diagnostic accuracy of the DDSS. Patients were found to frequently check their symptoms via an online platform with a minority using specific symptom assessment sites. Acceptability was also ‘good’ with most patients utilising Rheport, which is specific to rheumatology, rather than Ada which is more generalised [54]. In terms of diagnostic accuracy, the agreement was not promising concluding that algorithm or AI-based DDSS cannot replace the complexities of establishing a medical diagnosis [55]. The limitations of the studies conducted affected the generalisability of the studies, although they constitute high-level evidence in the form of RCTs. Issues identified arising out of DDSS use included decreased usability rating by older patients and the potential misuse of scarce hospital resources for the implementation of such systems without a determined benefit [55]. DDSSs can potentially replace online search engines for symptom assessment, providing specific advice on medical symptomatology; however, they require drastic improvement. Potential exists for integrating information gathered into patient EHRs and the incorporation of large language models, eg, Chat GPT to significantly improve performance [55]. A summary of studies involving DDSS can be seen in Supplementary Table S5, Appendix A.

### Cloud platforms

One cross-sectional study evaluated a cloud platform, PICASO (the PeopleSoft Intelligent Chat Assistant PICASO platform) on patients with RA [56]. Feedback was mainly positive from both patients and physicians with the cloud platform use (Supplementary Table S6, Appendix A). Some benefits included improved patient management, motivation, and communication. Challenges associated with use included technical difficulties, the need to adjust parameters once additional variables are to be considered, and issues regarding the lack of testing with other medical devices. The use of cloud platforms has potential for generating big data scenarios which can be used for research and development in the future.

## DISCUSSION

The integration of digital health technologies into rheumatology practice presents a fundamental change with the potential to revolutionise multiple areas of disease management from diagnosis and monitoring to therapeutics and patient empowerment [57]. This scoping review sought to provide an updated view of the existing evidence on digital health technology in rheumatology, highlighting both benefits and drawbacks, to inform future research and optimise patient care.

From this review, the benefits associated with digital health technology in rheumatology practice included the potential for enhanced disease management via continuous patient monitoring and personalised feedback mechanisms [[29], [30], [31], [32], [33], [34], [35], [36], [37], [38], [39], [40],[47], [48], [49], [50], [51], [52], [53]], increased patient engagement through interactive digital tools and readily accessible remote support networks [[41], [42], [43], [44], [45], [46], [47], [48], [49], [50], [51], [52], [53]], the improvement of access to rheumatological care [22,26,[42], [43], [44], [45],^47^,^50^,^52^,^53^], especially for individuals residing in remote or traditionally underserved geographical areas, and increased patient satisfaction [17,18,[41], [42], [43],^53^,^56^]. Challenges associated with the implementation of digital health technology in rheumatology practice, as revealed in the review, included a complex interplay of factors that transcended the limitations of patient-specific concerns. The heterogeneity in digital literacy among patients, varying from completely new learners to technologically advanced individuals, has emerged as a crucial factor influencing the successful adoption of digital health tools. Compounding this issue is the observed digital health literacy disparity amongst older adults [32,41,53]; this is further exacerbated by the apparent lack of dedication to examining the diverse factors influencing their ability to effectively engage with and benefit from DHIs. Disparities with patient access to technology have also been revealed as a major challenge affecting the implementation of such technology. Although digital health technology can potentially improve access to care for persons living in remote areas via the utilisation of wearable devices and telemedicine services, etc, patients especially living in remote areas with scarce technological resources, eg, internet connectivity, can pose a challenge. In addition, elderly patients with disabilities and patients with low socio-economic status may be prevented from accessing these technologies, contributing to the great technological divide [15], [20], [21], [23], [24], [25], [29], [32], [35], [39], [40], [41]. For physicians, the challenges associated with the implementation of digital health technology in rheumatology practice extend beyond mere adoption, necessitating comprehensive training programmes and readily accessible technical support systems to ensure the seamless assimilation of these tools into existing clinical workflows [41,42,44,45], thereby maximising their utility and mitigating potential disruptions to established patient care protocols.

The previous evidence synthesis derived from this study in Table 1 highlighted several gaps in the literature, underscoring the imperative need for additional, high-quality research to address the limitations and biases inherent in previous studies, and to further explore the efficacy, safety, and cost-effectiveness of various DHIs across diverse patient populations and clinical settings within rheumatology practice. The first systematic review investigating telemedicine [12] in Table 1 found telemedicine to be generally effective for the management of chronic rheumatological diseases, however, with limited evidence to support its effectiveness. In this scoping review, only 2 RCTs conducted showed improvement in patient outcomes with telemedicine as stated previously [16,17], whereas 2 RCTs [18,20] revealed no significant differences in patient outcomes with telemedicine. The RESULTAR study [25] revealed a consensus that telemedicine is more suitable for follow-up patients and that first-visit patients, as well as those patients with higher levels of disease activity, digital barriers, and cognitive impairment, should be attended to in person. The RESULTAR study was a large-scale, multicentre study allowing for generalisability, hence closing the evidence gap highlighted from previous evidence synthesis. A similar conclusion regarding telemedicine suitability for follow-up patients with lower disease activity was echoed in another study [20]. Of note, the 2022 EULAR points to consider for remote care in rheumatic and musculoskeletal diseases [58], which is a significant study which recommends that final diagnoses and initiation of DMARD therapy for patients with rheumatological conditions should be done via face-to-face visits. This aligns with the findings of previous evidence synthesis. There remains an evidence gap, however, regarding the assessment of the cost effectiveness of telemedicine as there were no studies in this review which investigated the same. The systematic review [13] highlighted ePROs as a growing area of interest in the field of rheumatology, which was confirmed in this review with 12 primary studies investigating its use. The use of mainly generic ePROs was also reflected in this review highlighting the need for future studies investigating disease-specific ePROs [13]. The finding of improved disease activity with ePRO use alongside mhealth technology from previous evidence synthesis was not adequately reflected in this study where 2 out of 3 RCTs did not result in improved disease activity with ePRO use [29,30]. In terms of the finding of ePRO correlation with physician reported disease activity or correlation between accurate identification of patient disease from previous evidence synthesis, the findings of this review are in agreement as 3 studies revealed such correlation and accuracy of ePROs in this aspect [33,37,38]. There was also a recommendation from previous evidence synthesis for future studies to assess ideal frequency of ePRO collection and potential for increased patient engagement [13]. Seven studies in this review had positive outcomes towards patient engagement with ePROMs [29], [30], [31], [33], [34], [35], [36], [40]. One multinational, multicentre RCT [32] concluded that a daily electronic diary can diminish recall bias and lead to significant improvements in the quality of PRO collection. The systematic review and meta-analysis [14] investigated eHealth and mHealth interventions in patients with JIA. The studies in this review investigating the impact of digital health technology in paediatric patients involved only patients with JIA of which the studies were already included in the previous evidence synthesis. There was therefore no additional evidence generated from this review on the impact of digital health technology on paediatric patients. The remaining evidence synthesis [8] and [15] explored big data, AI, and eHealth in chronic arthritis. Some of the studies from the previous evidence synthesis were also included in this review due to the time frame chosen within the last 10 years. Studies in this review largely agree that the preceding technology can be used for increased efficiency in the management of chronic rheumatological diseases. A study investigating the use of the PICASO cloud platform in patients with RA agrees that the generation of a big data scenario with AI components can open the door to evidence-based research across many disciplines [56]. It is important to note that all the previous evidence synthesis acknowledged the need for further research into the impact of digital health technology on conditions apart from chronic inflammatory arthritis where RA accounted for most of the conditions investigated. This review also reflected the same, where inflammatory arthritis in the form of RA was the most common condition studied which may be due to RA accounting for a higher number of patients presenting to rheumatology practice [59] and the condition having long-term established classification criteria and various validated disease activity scores.

It is noteworthy that most of the studies in this review were published after the introduction of COVID-19 to the world stage with n = 33 or 80.5% of primary studies being published from 2020 and beyond. Globally, the COVID-19 pandemic has been acknowledged as a significant driver behind the healthcare transition to digital transformation due to the increased need for remote monitoring and care [60]. This can explain most studies being conducted and published after the introduction of COVID-19. It is important to note that several technological advancements have been in limited use prior to the COVID-19 pandemic, eg, telemedicine/telehealth and some virtual care platforms [23,60].

A key takeaway is the major possibilities regarding the implementation of digital health technology in rheumatology care. This may include the generation of a big data scenario with increased linkages, allowing opportunities for evidence-based research and data collection in real time [37,40,55], the identification of patient subgroups which may benefit from more frequent care [34], and improved patient empowerment and communication.

### Limitations

Most of the studies in this review originated from Europe and the Americas which may have contributed to geographical bias. There may exist several explanations for these observances, which may include the distribution of rheumatologists and digital health technology globally, the native language of the countries included where non-English studies and databases that are not in English may have been overlooked, and the major global rheumatology societies being the European League against Rheumatism and the American College of Rheumatology. Europe and North America have been the global leaders in rheumatology for some time [61]. In this review, most of the studies from Europe originated in Germany n = 7, and 4 studies from the Americas originated from Canada which can be considered part of North America. Europe has also been considered 1 of the most digitally mature regions by the World Economic Forum [62]. Countries which may have had no studies published in the time frame of this review, eg, Africa, Oceania, and the Caribbean region may have either been due to a shortage of rheumatologists in the region, disparities with access to digital technology, language barriers resulting in publications in non-English Journals, or difficulties in accessing major journals that are mainly searched when conducting systematic reviews. For example, in the Caribbean region, there has been a documented shortage of rheumatologists [63,64], which would result in the lack of quality research into the impact of digital health technology in rheumatology care in that region. A study in Trinidad and Tobago [65] produced significant findings investigating telemedicine and patient satisfaction with rheumatology care; however, it was not included in the major databases outlined in the methodology and can provide further insight into reasons why studies from other world territories are not usually included in systematic reviews. These can include difficulties with publication fees from major English-speaking journals and the assumption of low relevance from editors associated with most high-end journals on studies from low-income to middle-income territories. In terms of limitations of the review methodology, this review was initially conducted as part of a master’s dissertation, where registration of a review protocol was not a requirement and, although not usually considered mandatory for scoping reviews, the potential overlaps in the definition of the various forms of technology may have proved difficult in registering a protocol. Another limitation was the inference that the articles examining the various forms of digital health technology would cover benefits, possibilities and challenges encountered for the various forms of technology without utilising deliberate search terms such as ‘benefits’, ‘challenges’, and ‘possibilities’. In addition, the notable methodological heterogeneity of the studies contained in this review and the potential overlap in categories used to describe DHIs are acknowledged limitations; hence, findings should be interpreted with caution.

### Implications for practice

Digital health technology has the potential to transform the landscape of rheumatology management through enhanced remote monitoring capabilities, facilitating the continuous assessment of symptom burden and functional limitations within the patient’s naturalistic environment [66]. Remote monitoring systems show promise in the early detection of acute exacerbations in individuals with decompensated chronic rheumatic conditions, which can facilitate prompt therapeutic adjustments and potentially mitigate the need for acute care interventions or hospital admissions [67]. In terms of implications for challenges regarding patient digital literacy level and access to technology in remote areas, there is a need for patient workshops to teach patients to utilise the various forms of technology in healthcare as well as infrastructural developments to ensure fundamental technological infrastructure, eg, internet access are available to persons residing in remote locations in order to maximise the use of digital health technology. The need for shared decision-making in the utilisation of digital health technology between patients and physicians is also paramount. The application of digital technology in rheumatology is rapidly expanding. This, together with the incorporation of AI, offers new avenues for improving diagnostics, personalising treatment strategies, predicting disease progression, and increasing opportunities for research [57,68].

### Recommendations for future research

Recommendations for future research would include additional RCTs to be conducted on the use of separate and specific forms of digital health technologies in rheumatology practice, eg, telemedicine/DHI applications to obtain more high-quality studies on patient outcomes, such as disease activity, hospitalisations, quality of life, and adherence to treatment. This will allow for systematic reviews whereby a meta-analysis can be done to strengthen the evidence base on the topic. High-quality studies investigating cost effectiveness of various technologies, as well as studies incorporating conditions apart from inflammatory arthritis, are also recommended. There is a need for increasing studies investigating digital health technology in the paediatric population. In addition, greater attention needs to be afforded to studies conducted with a view towards data privacy protections, equitable access, implementation of specific technologies, and regulatory certification, ensuring that emerging digital health technologies can be safely, effectively, and ethically incorporated into rheumatology practice.

## CONCLUSIONS

Digital health technology has potential value resulting in benefits to self-management with increased efficacy via remote monitoring, improvements in clinical care with high levels of patient satisfaction, and new research opportunities stemming from the ability to record patient data in real time. This can lead to health advancements at an individual and population level for both rheumatology practice and general healthcare, once issues identified arising from its implementation have been adequately addressed. Additional research of high quality is needed to strengthen the evidence base to implement policy changes incorporating digital technology in healthcare.

## Contributors

ER: Conceptualisation, methodology, investigation, resources, formal analysis, writing—original draft, and writing—review and editing. ES: Conceptualisation, methodology, validation, and supervision. All authors read and approved the submitted version.

## Funding

Not Applicable.

## Competing interests

The authors have no conflicts of interest to disclose.

## Patient consent for publication

Not applicable.

## Ethics approval

Not applicable.

## Provenance and peer review

Externally peer reviewed.

## Figures and Tables

**Table A1:** A Summary of Studies involving Telehealth and Telemedicine

Study ID	Design	Technology & Aim	Outcome	Challenges	Future Implications
Salaffi *et al***.** 2016 [16]	Single site RCT (Italy)	To investigate whether a telemonitoring intensive strategy (TIS), is more effective than conventional strategy in reaching remission and obtaining disease control in early RA patients.	A higher percentage of patients in the TIS group achieved CDAI remission vs patients in the conventional strategy group for RA patients (38.1 % vs 25 % at year 1, p <0.01).	The study was limited to patients who were able to use the internet which limited generalizability.	Participating rheumatologists indicated that the telemonitoring system could be a helpful tool in daily clinical practice.
			Time to achieve remission was significantly shorter in the TIS group		
			The TIS group showed greater improvement of functional impairment (71.4 % vs 35 %) and radiological damage progression (23.8 % vs 10 %), resulting in a greater rate comprehensive disease control (19.4 % vs 5 %).		
Taylor-Gjerve et al 2017 [18]	Multicentre RCT (Canada)	To evaluate whether rheumatoid arthritis (RA) patients using video-conferencing and inter-professional care support have comparable disease control to those utilizing traditional in-person rheumatology clinics.	No significant between‐group differences in disease activity, quality of life or patient satisfaction scores were noted compared to traditional in-person visits for RA patients	Technical difficulties arose during 10 video‐conferencing visits, resulting in only an audio‐link being available.	Strategies for the development of models of care need to involve consultation with the people they are being developed to serve
			Satisfaction rates were high in both groups: the intervention group rated their care as “excellent” more often (76%) than the control group (59%).	Whilst distance technolology can be beneficial, there may be a loss of auxiliary benefits for others which the travel facilitates/supports	Video‐conferencing in an inter‐professional collaborative care model appears to be feasible for stable RA patients living in remote areas
Ramlet *et al***.** 2017 [17]	Single site Randomized Crossover Trial (Switzerland)	To evaluate the impact of a nurse-led telephone intervention or Telenursing (TN) compared to standard care (SC) on satisfaction and health outcomes of children with inflammatory rheumatic diseases and their parents.	Satisfaction was significantly higher when receiving TN (OR = 7.7, 95% CI: 1.8–33.6)	Participants receiving the TN first were likely to require more support and help than those who received the intervention minimum one year after diagnosis.	This study highlights the importance of support by specialist nurses in improving satisfaction and symptom management for children with inflammatory rheumatisms and their families
			Morning stiffness (OR = 3.2, 95% CI: 0.97–7.15) and pain (OR = 2.64, 95% CI: 0.97–7.15) were lower in the TN group. For both outcomes a carry-over effect was observed with a higher impact of TN during the 12 first months of the study.		Newly diagnosed patients with complex needs require close monitoring and time to adjust to the diagnosis, particulars of treatment, and to cope with doubts and future uncertainty.
Kong et al 2021 [25]	Cross Sectional Study (Florida, USA)	To assess patients’ attitude on the perceived effectiveness when comparing TM to in-person visits, as well as patients’ access to technology, distance travelled by the patient to attend the clinic visit, and demographic parameters.	Rheumatology Clinics are increasingly turning to Telemedicine	Access and familiarity with technology may still be limited	Studies needed focusing on aiding elderly patients to utilize TM services
			Patients with a greater commute preferred TM consultation.	Increased difficulty with elderly patient populations in utilizing TM.	Guidelines need to be established regarding TM use in outpatient clinics.
			Follow-up patients were more likely to feel that their visit could have been possible over the phone or video conferencing compared to new patients	Younger patients reported more conflict between appointments and work hours.	
George *et al***.** 2021 [22]	Multicentre, Retrospective cohort Study (USA)	To investigate the impact of COVID19 on rheumatology care delivery in a large community practice–based network and the impact of telehealth in continuity of care	COVID-19 led to large disruptions in rheumatology care which were only partially offset by increases in telehealth use.	Telehealth disproportionately affected racial/ethnic minorities and patients with lower socioeconomic status.	Telehealth approaches need to be made more accessible, irrespective of the important social determinants of health that impact access to technology-enabled care.
			Patients with certain autoimmune rheumatic diseases (e.g., RA, psoriatic arthritis, systemic lupus erythematosus) were less likely to cancel visits during the COVID-19 transition period compared to patients with gout, osteoarthritis, and osteoporosis, possibly reflecting the need for close monitoring among patients with certain autoimmune conditions.	Within a large, multistate, community rheumatology practice network, follow-up visit cancellations were as high as 60% at the height of the COVID-19 pandemic and were more common in patients who were older, Black, Hispanic, of lower socioeconomic status, and residing in rural areas.	Collecting disease-specific electronic patient-reported outcomes using a digital platform via a smartphone app and/or passive monitoring device may also be useful complements to delivering high-quality remote patient care.
			Telehealth grew from almost no use to >40% of follow-up visits at the height of the COVID-19 pandemic, but older age, lower socioeconomic status, and rural residence were associated with lower use of telehealth.		Interventions to improve access to and/or assistance in using telehealth technology is particularly important for vulnerable populations.
Naveen *et al***.** 2021 [23]	Prospective Cohort Study (India)	To describe the teleconsultation experience in a prospectively followed cohort of adult and juvenile IIM.	151 teleconsultations were sought over a 93-day period by 71 (52.2%) of 136 IIM (median age 38 years, F:M 4.5:1). Nearly one-third (38%) consulted on an emergency basis, with voice consultations being the primary medium of communication.	Patients who relapsed required more consultations [2(2–3) vs 1(1–2), p 0.009].	Voice-based teleconsultations may be useful to diagnose and manage relapses in IIM during the pandemic. Patient education for meticulous and timely reporting may improve care.
			Over a quarter (26.8%) reported relapse (15.5% minor, 11.3% major), these being more common in JDM [71.4%, OR 8.9 (1.5–51)] as compared with adult IIM, but similar across various IIM subtypes.	Challenges encountered included outdated phone numbers, recording outcome measures, language and communication barriers, poor quality and lighting while taking pictures and videos, dealing with the technology gap and accessibility to laboratory services.	Larger multicentre studies may identify subsets of IIM that require greater care and early tele-triage for effective management of the condition.
			The demographic and socioeconomic profile of the patients seeking consultation (n = 71) was not different from those who did not (n = 65).		Tele-care may be harnessed with the vision to follow tele-triage, advance telesorting to ease rush of cases post-pandemic, formulate community outreach through a structured referral system, and deliver ancillary care.
Ferucci *et al***.** 2022 [19]	Single site cross sectional study (Alaska)	To evaluate outcomes and quality of care for rheumatoid arthritis (RA) in patients seen by video telemedicine follow-up compared to in-person only	In short-term follow-up, there was no significant difference in most outcome and quality measures in patients with RA who incorporated telemedicine follow-up in their care compared to in-person only.	The study provided a comparison of telemedicine incorporated into practice along with in-person care, not telemedicine alone, due to existing practice constraints	Telemedicine can reasonably be offered as a component of care for RA and may provide the ability for patients to be seen more often.
			In univariate analysis, functional status improved over 12 months in the telemedicine group.	A randomized controlled trial of telemedicine is no longer feasible in most practice settings, given the increase in use during the COVID-19 pandemic and sustained use in the post pandemic period.	Benefits of telemedicine e.g. cost reduction and improved communication, may make it attractive even if outcomes have not improved compared to in-person only care.
			In multivariate analysis, RAPID3 and functional status were associated with telemedicine group (higher), with no statistically significant change over the 12-month period.		
Sloan *et al***.** 2022 [21]	Mixed Methods (Multisite, UK)	To investigate rheumatology patient and clinician experiences and views of telemedicine	Patients and clinicians rated telemedicine as worse than face-to-face consultations in almost all categories, although >60% found it more convenient.	Telemedicine was perceived to have increased misdiagnoses, inequalities and barriers to accessing care. Participants reported highly disparate telemedicine delivery and responsiveness from primary and secondary care.	Training, choice, careful patient selection, and further consultation with clinicians and patients are required to increase telemedicine’s acceptability and safety.
			Telecare was noted to be more useful for patients in remission.	Double working for clinicians, e.g. double scheduling for both teleconsults and physical visits in cases of patient flares Spending hours replying to patient emails.	
So *et al***.** 2022 [20]	RCT (China)	To evaluate patient satisfaction, compliance, disease control, and infection risk of telemedicine (TM) compared with standard in-person follow-up (FU) for patients with lupus nephritis (LN) during the COVID-19 pandemic.	TM FU resulted in better patient satisfaction and similar short-term disease control in patients with Lupus Nephritis compared to standard care.	Patients in the TM group was associated with more hospitalizations.	Data suggested TM was equally efficacious in maintaining disease control in patients with LN in short-term, although it might need to be supplemented by in-person visits, especially in patients with higher physician assessed disease activity.
			There was a higher physician global assessment score (PGA) in the TM group.		
Rubio *et al***.** 2022 [24]	Single-site, Retrospective, cohort study (Spain)	To analyse the demand for rheumatology consultations from Primary Care and their resolution using the electronic consultation system	Over 40% of queries were resolved due to the electronic consultation system in an average of 48 hours, otherwise patients would have been referred to specialized care.	Those for whom procedures (arthrocentesis or ultrasound) needed to be performed or in whom the diagnosis is complex were also candidates.	E- consultations avoids unnecessary patient movement within the system and provides an alternative response to increased demand, acting as a triage system, reducing waiting times.
			Waiting time is reduced, with quicker reception of a response, allowing faster access to the outpatient circuit if a preferential or urgent assessment is required		Triage systems should be set up in which patients with stable diseases and well-established diagnoses or with small flare-ups that can be managed with minimal changes in treatment would be candidates for telemedicine consultation
RESULTAR study, Gil *et al***.** 2024 [26]	Cross-sectional, multicentre study (Spain)	To analyse the application of telemedicine in rheumatology consultations	TM is useful for follow-up of some patients, to help determine if face-to-face consultations are necessary	There was no consensus as to whether TM improves the quality of the service provided, establishes closer and more personalised communication, or that it promotes healthcare irrespective of the resources available.	A consensus was achieved that first appointments and those of individuals with active inflammation, digital barriers, sensory difficulties, or cognitive impairment could be attended to in person.
			TM can assist patients with rheumatoid arthritis if they present low activity or in remission	95% of the respondents felt that data security systems for TM will need to be put into place.	TM can be a valuable tool for monitoring patients with rheumatic diseases, as well as alleviating the burden of face-to-face care in rheumatology
			Patients in their first consultation or those who present digital barriers or cognitive deterioration, should be seen face-to-face	Some of the main limitations in the use of TM were specifically administrative/ technical, the lack of suitable equipment, or poor internet connection.	
			TM is useful for alleviating the face-to-face care burden in rheumatology		
Zafar *et al***.** 2024 [27]	Multicentre, Qualitative Study (Canada)	To explore perspectives and experiences of health care providers (HCPs) and patients related to the use of WelTel, a text messaging support program, as an adjunct to routine care. for RA patients	Patients’ views suggested that text messaging with the rheumatology team supported high-quality care across multiple quality domains including patient-centeredness, timeliness, efficiency, safety, effectiveness, equity, and appropriateness.	The impact of increased communications on HCP burnout and workload requires consideration.	Factors to consider for implementation of text messaging include further development and evaluation of interdisciplinary care models and including administrative staff to ensure messaging is addressed by the most appropriate individual.
			HCPs’ perspectives varied based on their experience with the WelTel platform. Adopting providers perceived texting as providing high quality care, whereas nonadopting providers had concerns about security of information, effectiveness, efficiency, and raised health equity concerns with the technology.	Perceived limited digital literacy of older patients, communication barriers for those with limited English proficiency, and the inability of those unable to afford a cell phone were challenges to partake in this method of care.	
Zickuhr *et al***.** 2024 [28]	Online, Qualitative Study (Washington USA)	To explore the benefits and shortcomings of shared decision making (SDM), regarding chronic rheumatological conditions during Virtual Care (VC)	SDM is an essential component of health care, supporting patient autonomy, medical adherence, and health equity in patients with rheumatologic and chronic conditions (RCCs).	Virtual care (VC) expands access to patients with RCCs, yet studies suggest SDM is reduced during VC.	VC can enhance SDM by giving patients with RCCs more time to make choices and providing alternate avenues for patient education.
			VC, a mainstay of care delivery, provides a virtual platform for SDM while also offering solutions for barriers to SDM.	Emotionally charged decisions, new diagnoses, and severe or end-stage conditions are better suited for in-person SDM	SDM occurs successfully when VC skills, such as communication, interpersonal relationship building, and constructing health systems, support the steps of SDM during virtual encounters and when patients and providers can implement these skills virtually.
				Unstable, insecure, or no access to internet or telecommunications.	

CDAI: Clinical Disease Activity Index; RCT: Randomized Controlled/Crossover Trial; IIM: Idiopathic Inflammatory Myopathy

**Table A2:** A Summary of Studies involving Electronic Health Records and Electronic Patient Reported Outcome Measures

Study ID	Design	Technology & Aim	Outcome	Challenges	Future Implications
El Miedany *et al***.** 2016 [29]	RCT (London, UK)	To assess the use of ePROM, and its ability improve disease activity in patients with early RA	There was no significant difference between disease activity measures as well as DAS28 and RAPID-3 scores at 3, 6, and 12 months of management.	The integration of the ePROM system into standard care requires significant investment of resources and planning.	ePROM can also be used as a performance index for RA patients in addition to its role in clinical practice.
			There was a trend toward lower patient-reported tender joint count and functional disability score in the active group versus the control group.	Developing a new ePROM system requires a significant amount of programming time as well as dedicated project management and leadership.	
			Patients’ adherence to antirheumatic therapy was significantly higher (p < 0.01) in the ePROM group, whereas stopping disease-modifying antirheumatic drugs for intolerability was significantly higher (p < 0.01) in the control group at 12 months of treatment.	Getting doctors/nurses on board remains a challenge.	
Bingham *et al***.** 2019 [34]	Multicentre, Multinational RCT	To assess the implementation of ePROs in patients with RA	High compliance rates support the use of the daily electronic PRO diary in large RCTs, (RA-BEAM 94% patients; RA-BUILD 93% patients).	Identified process challenges included non-timely issuance of the device, low battery, inadequate training of patients before data collection, inappropriate diary set-up, and first response entry 1 day after the baseline visit.	The daily electronic diary is expected to reduce recall bias and improve the quality of PRO data collection.
				The study team learned that there should be a more robust process for tracking lost or defective devices.	Capturing and reporting more reliable PRO data, collected in real time, can provide further evidence of beneficial treatment effects on outcomes.
Zhao et al 2020 [32]	Multicentre Cohort Study (USA)	To develop classification algorithms that accurately identify axSpA patients in EHRs and compare the performance of algorithms incorporating free-text data against approaches using only ICD codes.	Classification algorithms incorporating narrative EHR data and machine learning can accurately identify evolving disease concepts such as axSpA compared to using ICD codes only.	Performance of these methods depends on the diagnostic codes accuracy, which can vary substantially across healthcare systems.	Large cohorts identified using these novel methods via EHRs offer exciting opportunities for future clinical research even with evolving disease definitions and can be used to study healthcare utilization or rarer outcomes overlooked by prospective registries.
			The automated MAP algorithm allows large cohorts of even uncommon diseases to be efficiently created, offering exciting opportunities for future clinical research.	ICD codes may not be well defined for evolving disease concepts such as axSpA where there are no specific codes up to the 10th version.	Algorithms incorporating free-text data improve the accuracy for classifying axSpA patients in electronic health records.
				Not all EHR systems may be able to provide data used in our development process, such as word searches in the initial enrichment process.	Further studies are needed to externally validate the axSpA algorithm used in different EHR systems and other countries.
Liu *et al***.** 2021 [39]	Multicentre Qualitative Study (USA)	To assess patient and physician acceptance of a patient-facing dashboard visualizing PROs for patients with RA	Patients and clinicians agreed that the dashboard could enhance communication about PROs and RA disease activity and could promote patient self-management.	Clinicians expressed significant concerns about the logistics of using the dashboard in clinical practice.	Additional varied dashboards can be more effective—one for physicians with more detailed information and another for patients that is more limited.
			The ability to customize the data display is important for tailoring the dashboard to patients with diverse needs and preferences.	Many patients found the dashboard difficult to understand at first glance.	Special attention should be given to feasibility concerns voiced by clinicians.
					Training videos or tutorials may need to be developed to enhance patient understanding of the RA dashboard.
Lee *et al***.** 2021 [30]	Single-site RCT (USA)	To examine the effects of a digital health application to monitor ePROs on patient satisfaction and disease activity in patients with RA	A mobile app designed to collect ePRO data on RA symptoms did not significantly improve patient satisfaction or disease activity compared to care coordination alone.	A “one size fits all” approach is unlikely to be efficacious, making it critical to precisely define the target study population.	Identification of barriers to implementation, quantitative assessments of each step are necessary.
			No statistically significant group differences in 6-month medians of TSQM, PEPPI, or CDAI were detected.	Many digital intervention studies require substantial physician involvement which may require knowledge in areas in which physicians do not have expertise (e.g., interpretation of ePROs).	Resources must be provided to educate physicians on these measures and methods to integrate electronically generated information into their workflow.
			Of the 67 intervention participants who completed the exit survey, 90% rated their likelihood of recommending the app as ≥7 out of 10. Of the 11 physicians, 73% agreed/strongly agreed that they wanted to continue offering the app to patients.		Identifying the needs of specific subgroups of patients and designing the app such that it can be tailored to specific situations are necessary for smartphone application success.
Muehleisen *et al***.** 2022 [35]	A qualitative study embedded in a multicentre RCT (Germany)	To investigate patient and physician experiences, perceived drawbacks and benefits of using an ePRO web-app (ABATON RA) to digitally support shared decision making, (SDM) and treating to target, (T2T)	Patients and physicians appreciated having an improved overview of disease activity, capturing disease flares and continuous remote monitoring.	Certain ePRO seemed outdated and were hard to understand.	Physicians reported that app-based continuous documentation of ePRO could be helpful in other medical domains, such as multiple sclerosis, heart failure, chronic kidney disease, diabetes etc.
			Paper and timesaving were associated with using ePRO.	Physicians feared becoming too focused on collecting ePRO data and stressed the lack of reimbursement for monitoring ePRO and lack of app interoperability.	Study participants of both user groups reported that the approach trialled could improve current rheumatology care.
			For RA patients and physicians, benefits of the digitally supported SDM using ePRO seemed to outweigh observed drawbacks.	Persons lacking in technical skills, health literacy would be left out.	
			Physicians emphasized that app utilization can promote time savings in patient care.		
Nasrallah *et al.* 2024 [36]	A qualitative study embedded in a single centre RCT (TELERA) Germany	To explore experiences of RA patients and rheumatology staff regarding the new care model involving a digital app to record ePROs.	Participants described current follow-up care as burdensome with patients in remission having to travel long distances.	Financial and psychological barriers were identified among rheumatologists preventing them from cessation of automatically scheduling new appointments for patients in remission.	To ensure success, the model needs to be more responsive and allow seamless integration of education material.
			Patients reported that using all study components (medical app for ePRO, self-performed CRP test and joint self-examination) was easy and helped them to better assess their disease condition.	Rheumatology nurses pointed to the potential lack of personal contact, which may limit the holistic care of RA-patients.	
			It was perceived that the new care model enabled more patient autonomy, allowing patients more control and flexibility at the same time. All components were well accepted and easy to carry out for patients.	Patients wanted to be automatically contacted in case of abnormalities or to have an app feature to request a call-back or chat.	
				Participants homogeneously reported the enormous workforce shortage as the main challenge in rheumatology care.	
				Patients had to know certain terms and vocabulary to use the medical app and perform joint self-examination.	
Uhrenholt *et al***.** 2022 [33]	Single-site Randomized Cross over Trial (Demark)	To evaluate the agreement of PROMs between the DANBIO app and outpatient touchscreen in patients with IA.	In patients with IA, equivalence was demonstrated between two device types for all PROMs except BASDAI; however, BASDAI was within the limits of the minimally clinically important difference, MCID.	The patient population was largely restricted to patients with well-controlled disease activity; therefore, the variability in PROMs for patients with low disease activity may be less than for patients with high or moderate disease activity.	Implementation of the DANBIO app is expected to help physicians to prioritize outpatient visits, thereby improving the management of IA both for the individual patient and for society.
Bell *et al***.** 2022 [37]	Multicentre, Multinational RCT	To assess the compliance with a phone application and validate ePRO information in individuals with systemic lupus erythematosus (SLE).	The excellent compliance (>75%) and the high level of consistency between data collected by paper and that collected by electronic methods (73.4%) indicate that the app provides a reliable means of cataloguing real-time changes in PROs in SLE patients.	One issue with current instruments is that PROs are often recorded intermittently, typically during an in-person clinic visit, and require patients to recall a period of several weeks or months.	The development of mobile technology for dense electronic patient-reported outcome (PRO) data collection allows for routine assessment of systemic lupus erythematosus in real time and in the patient’s regular environment.
			The data suggest that PRO collection by app could replace that done in the clinic by paper or electronic methodology.	It is uncertain whether the app will work comparably in general practice with patients of varying health literacy profiles.	High compliance with phone application usage and consistency between reporting methods offers immediate access to reliable PRO information.
				There was some manifestation of app fatigue in this trial, in the future this might be mitigated by rewards, simplification, or providing patient access to their personal data.	The acceptability of apps can now be assessed with rating scales, such as the Mobile Application Rating Scale (MARS) that could be useful to evaluate the app more fully.
Li et al 2023 [31]	Multicentre open label RCT (China)	To determine whether utilizing a digital health application to assess PROs can lead to achieving disease control in patients with RA.	At month 6, the rate of patients with DAS28-CRP of 3.2 or less was 71.0% (780 of 1099 patients) in the SSDM group vs 64.5% (708 of 1098 patients) in the control group (difference between groups, 6.6%; 95%CI, 2.7%to 10.4%; *P* = .001).	Cluster randomization is a more appropriate design due to the minimization of communication between patients as well as modification of physician behaviour, which make it harder to get significant effects.	Application-based patient-reported outcomes and intervention could be an effective way to treat patients with RA and may provide evidence for diseases with complex treatment targets.
			At month 12, the rate of patients with DAS28-CRP of 3.2 or less in the control group increased to a level (77.7%) that was comparable with that (78,2%) in the SSDM group (difference between groups, −0.2%; 95%CI, −3.9% to 3.4%; *P* = .90).		The SSDM system has the potential to serve as a supplementary platform for reporting adverse events, confirming the findings of previous research.
			The use of a digital health application with patient-reported outcomes was associated with an increase in disease control rate.		
Nasrallah *et al***.** 2024 [40]	Single-site Qualitative Study (USA)	To assess clinician perceptions and experiences using the RA PRO dashboard in a university rheumatology clinic.	The dashboard showed promise in enhancing patient-clinician communication, shared decision-making, and overall acceptance among clinicians.	Inconsistent collection of RA outcome measures, particularly during virtual visits from the pandemic period, was a common issue mentioned by clinicians.	Future work should attempt to quantify the specific impacts that dashboard use has on long term disease outcomes, shared decision making, patient satisfaction, adherence to treatment, and costs of care.
				Additional training for administrative staff, and even patients towards emphasizing the routine collection of outcome measures may be required.	Further research and ongoing improvements in dashboard design and implementation are warranted to ensure its successful integration into routine clinical practice.
				Insufficient data populating on the dashboard, including missing historical scores, can limit the dashboard’s utility	
Thomassen *et al***.** 2024 [38]	Cohort study utilizing RCT data (Norway, Northern Europe)	To assess axSpA patients’ willingness to use remote care and adherence to reporting of ePROs	The majority (96%) reported high willingness to use remote care.	Many patients needed the automatic reminders throughout the study period in order to remember to complete the ePROs.	Identification of potential patient subgroups with low or high adherence to reporting of ePROs may support healthcare providers in identifying patients that may benefit from remote care versus continuation of consultations.
			Adherence to reporting ePROs remained high over 18 months by remote monitoring and patient-initiated care groups 88% vs. 83%	The increased workload introduced by managing alerts, could potentially act as a barrier among healthcare professionals for future implementation of remote care.	
			The high degree of willingness and adherence to reporting ePROs over time indicates that the majority of axSpA patients with low disease activity are motivated to use remote care.	Technical problems could be another possible barrier for future utilization of remote care e.g. app malfunction.	
				Persons who reported using electronic or online resources expressed concerns regarding understandability and credibility of online sources.	

axSpA: axial spondyloarthropathy; CDAI: Clinical Disease Activity Index; DAS-28: BASDAI: Disease Activity Score-28; EHR: electronic health record; ePRO/M: electronic patient reported outcome/measures; IA: Inflammatory Arthritis; ICD: International Classification of Diseases; PEPPI: Patient-Physician Information Exchange Questionnaire; RAPID-3: Routine Assessment of Patient Index Data 3; RA: Rheumatoid Arthritis; TSQM: Treatment Satisfaction Questionnaire for Medication

**Table A3:** Showing studies involving Digital Health Intervention (DHI), apps

Study ID	Design	Technology	Outcome	Challenges	Future Implications
Mason *et al***.** 2020 [46]	Qualitative Study (Leeds, UK)	To determine potential barriers and facilitators to the use of a DHI for patients with painful DUL MSK disorders	Both HCPs and people living with a DUL‐MSD welcomed the development of a DHI. Rewards and achievements were regarded as incentives to use a DHI.	Some participants were sceptical about digital management of DULMSDs and were not satisfied that the same standards of care could be offered digitally.	Attention needs to be paid to digital design features, usability, tailoring, personalisation and consideration of how well usual care could be replicated digitally without direct HCP involvement.
			The ability to review data to track and monitor progress was the most appreciated feature. The most used and most satisfactory functionality was communication with health professionals.	HCPs noted that some patients may be reluctant to engage with DHIs and could feel discouraged or ‘fobbed off’ when signposted to a digital programme instead of receiving usual care.	There is a need to ensure that people are empowered to participate in their own care and a DHI may provide this.
			Ease of use was recognised as vital in enabling a variety of users to engage with DHIs.	HCPs believed that a DHI could not replicate certain aspects of in person usual care.	Support from health professionals is key to increasing longer term engagement with these tools
			Most participants with DUL‐MSDs thought that users should be able to personalise and filter online content for their own individual needs.	Concerns were raised by participants with DUL‐MSDs about the consequences of following an unsuitable digital exercise programme.	
			DHIs were considered by participants with DUL‐MSDs to have high usability if they were perceived as credible, safe and secure.	Some HCPs expressed concerns about patients not performing the exercises correctly without someone to demonstrate and address any issues	
			The satisfaction survey yielded a median (range) score of 9.1 (7-10) out of 10. Retention was also high.	It was considered important that HCPs were aware of their patients' progress and not having HCP involvement may be a barrier to digital health engagement.	
				The supervisory role of healthcare professionals with these apps can increase their workload.	
Romero-Jimenez et al 2022 [45]	Prospective cohort study (Location undefined)	To assess the eMidCare® app’s impact on medication safety, communication, satisfaction, and usability in IMID patients starting biologic therapy	In the My Medication module, 100% of patients registered their biological therapy and 25.9% also used this module to record each dose of medication administered. A total of 82 adverse events (AEs) were registered. Thirty-two percent of the patients registered at least 1 AE.	No published studies on the usability of apps for IMID patients were found at the time.	Apps involving active participation of patients by data registration, messages, or gamification can improve usability.
			The most used and most satisfactory functionality was communication with health professionals. 52% of patients used the Messages module to communicate with healthcare professionals.	The supervisory role of healthcare professionals with these apps can increase their workload.	Support from health professionals is key
			The satisfaction survey yielded a median (range) score of 9.1 (7-10) out of 10. Retention was also high.		
			Best rated features were the ability to communicate with healthcare professionals, ease of app use, and reminders to administer medication.		
Pouls *et al***.** 2022 [44]	Multicentre RCT (GAMER STUDY) Netherlands	To assess the effect of adherence to DMARDS with an eHealth gaming intervention in RA patients	A serious game aimed at reinterpreting attitudes toward medication failed to show an effect on adherence to DMARDs or clinical outcomes in patients with RA.	Targeting implicit attitudes using eHealth has shown to be effective in changing health behaviour but has not been applied to medication taking behaviour for reasons possibly related to unchanged implicit behaviour.	The game was played frequently indicating that it can be an effective channel for reaching patients.
			Adherence in the intervention group (63%) and control group (54%) did not differ significantly (p=0.13) at 3 months, neither were there differences observed in CQR continuous score, beliefs about medication (BMQ) or clinical outcomes (HAQ and RADAI).	The serious game was not integrated in the RA care pathway and operated independently of the care context.	Combining eHealth interventions with healthcare professional interaction can increase the chances of intervention effectiveness.
Labinsky et al 2022 [41]	Single-site Cohort Study (Germany)	To evaluate the adherence, acceptance, and efficacy of a digital health application DTx, DiGA in patient with rheumatic diseases	48 patients were prescribed DiGA. A total of seven different DTx (DiGAs) were prescribed. Of these, 39/48 (81%) completed the follow-up survey. 21/39 (54%) patients downloaded the DTx and 20/39 (51%) used the DTx at least once.	Lack of time and commitment were reported as the main reasons for non-use	With high rates of attrition, exploring the determinants of adherence and utility of patient education in advancing digital health approaches and overcoming individual barriers for facilitating equity in health access for rheumatology patients is prudent.
			9/39 (23%) of patients stopped quickly afterward and 5/39 (13%) reported having completed the whole DTx program.	Others reported trying the DTx but encountered barriers to obtain the applications, among them technical issues such as lack of storage space, and outdated hardware or software.	
			Overall acceptance of DiGA was high (Net promoter score (NPS) mean (SD) 7.8/10 (2.3)).	The lack of commitment in the application, for example, no fixed dates (as for face-to-face appointments), was highlighted negatively and seen as a reason for infrequent use.	
			While the majority of patients (60%) reported no improvement, one subgroup of patients (7/20, 35%) who regularly used an exercise-based DTx for back pain reported symptom improvement.		
Shao *et al***.** 2024 [42]	Multicentre RCT (Taiwan)	To assess the effectiveness, feasibility, and acceptability of a smartphone app for adults with rheumatoid arthritis.	Outcome measures for the intervention group improved significantly more than the control group for self-efficacy of pain at 8 weeks (B = 6.39, p < .05) and for both of self-efficacy of pain and self-management behaviour at 12 weeks (B = 9.16, p < .05, and B = 4.25, p < .001, respectively) for RA patients.”	Challenges relating to technology literacy limited the usability of the app for some.	A greater understanding of how to overcome technological challenges is needed to optimize the delivery of self-management programs via smartphone-based apps.
			Patients expressed appreciation for having an indicator of improvement over the course of the intervention.	Familiarity with using a smartphone was a condition of participation	Studies re-evaluating this app on a larger sample size should be conducted
			Factors related to app ease of use included limiting pop-ups and windows and having no time restrictions.	Participants regularly forgot how to use the app and needed to ask family members for help and reported that this challenge caused their infrequent use of the app.	Holding a workshop to help all patients become comfortable with using the smartphone app may effectively reduce the perceived technological challenges of app use.
Blaskowitz *et al***.** 2024 [43]	Single site RCT (Germany)	To investigate the clinical effects of the ViViRA app compared with those of standard physiotherapy in patients with spondyloarthropathy	The intervention group showed a significant improvement in mobility using the BASMI score, p = 0.05, whereas the control group showed a significant decrease in mobility for the same score in patients with spondyloarthropathies, p = 0.00).	While the group practicing with ViViRA demonstrated positive outcomes, the physiotherapy group experienced greater improvement in perceived disease activity than did the intervention group (p = 0.03).	Existing research describes the improvement of therapeutical adherence through the design of applications. In ViViRA, messages with reminders and social support have a positive effect
			The intervention group demonstrated lower pain intensity (VRS pain level at week 3.5 ± 2.8) than did the control group (VRS pain level at week 4.5 ± 2) after 12 weeks.	A disadvantage of exercising at home with ViViRA is rooted in the lack of personal support. The patients do not get feedback and there is no control over their therapeutic adherence.	For a better analysis of the longitudinal effects, further studies with longer observatory periods are needed.
			Most participants in the intervention group desired to have the application re-prescribed which advocating for good adherence.		

BASMI: Bath Ankylosing Spondylitis Metrology Index; CQR: Compliance Questionnaire for Rheumatology DUL-MSD: Distal Upper Limb Musculoskeletal Disorder; DTx: Digital Therapeutic Application; HCP: Healthcare Professional; HAQ: Health Assessment Questionnaire; IMID: Immune Mediated Inflammatory Disease

**Table A4:** Showing Studies Involving Self-Monitoring and Assessment Tools

Study ID	Design	Technology & Aim	Outcome	Challenges	Future Implications
Gossec *et al***.** 2018 [47]	Multicentre RCT (France)	To assess the efficacy and access to an ehealth self-assessment website Sanoia® on patient-physician interactions for patients with RA	Giving RA patients access to the interactive Sanoia e-health platform led to a small improvement in patient-perceived patient-physician interactions (PEPPI-5 questionnaire)	A disjunction between patient satisfaction and access to the platform was noted.	eHealth platforms are promising in RA.
				Although mean satisfaction with the platform was very high, around a quarter of the patients did not use the interactive platform. 12% of patients expressed was that the platform was not useful because they were in disease remission.	Future studies of e-health should measure not only patient satisfaction but a combination of both patient satisfaction and utilization.
Conelly *et al***.** 2019 [48]	Multicentre RCT (USA)	To evaluate the efficacy of a Digital Self-Management Programme to improve pain and HRQL in patients with JIA	Primarily self-directed online self-management training and online disease education comparably and modestly improve pain and HRQOL in youth with JIA.	Adherence in the control group was higher than in the intervention group which was unaccounted for 73% vs 82 % respectively.	Future studies should consider directly exploring the optimal “dose” of therapist contact that maximizes benefits for eHealth interventions, and whether this varies as a function of patient demographic or clinical characteristics.
				The effect sizes were small and positive expectancy of an intervention may have affected the results.	Further research can determine other treatment features that enhance the effectiveness of online self-management interventions for youth with chronic conditions.
Stinson *et al***.** 2020 [49]	Multicentre RCT (Canada)	To evaluate the effectiveness of a self-assessment web based digital health application, (Teens Taking Charge) on symptom improvement and HRQL in JIA patients	The Teens Taking Charge Web-based intervention is effective at reducing both pain intensity and pain interference, as well as improving HRQL in adolescents with JIA, compared with education control. These effects are sustained for up to 12 months following program completion.	The website platform did not support the capture of user-level usage analytics at the time of development. Thus, the impact of program usage level on outcomes could not be examined.	The Teens Taking Charge program is now available at no cost. Efforts are underway to widely disseminate this resource to increase the accessibility of self-management care for adolescents and families living with JIA.
			Significant overall reductions in pain intensity (*P*=.02) and pain interference (*P*=.007) were observed for intervention group participants. There was a significant overall improvement in HRQL related to problems with pain (*P*=.02) and problems with daily activities (*P*=.01).	The higher rate of loss to follow-up in the intervention vs control group may have been the result of increased participant burden (eg, greater time commitment required to review intervention content vs control).	Efforts are also underway to culturally adapt the intervention for use in different settings.
Magnol *et al***.** 2021 [53]	Observational, multicentre cross-sectional study (France)	To describe eHealth usage and expectations in patients with RA	82.2% of the participants had access to eHealth. 86.1% through a computer, 40.2% via a tablet and 47.3% via smartphone.	Older patients use eHealth less often, and pain and joint deformations can restrict the use of eHealth devices among these patients.	Further studies need to be conducted to evaluate the reasons regarding the use and non-use of eHealth by patients with RA.
			All patients (100%) used eHealth tools to obtain information about RA, and 66.4% of them used it to monitor their rheumatism.	Lack of information provided by health professionals on various eHealth tools available and the discrepancy of eHealth use by physicians can result in its lack of promotion.	The use of a reliable and validated eHealth tool for rheumatoid arthritis could be promoted by rheumatologists and could optimize therapeutic adherence.
			Factors such as age, education level, employment, DMARDs, level of comorbidity, membership of a patient association, and participation in a patient education program were found to be associated with the use of eHealth tools for RA.	Lack of evidence-based medicine and concerns surrounding data security may affect usage of these tools.	
			68.2% of patients reported they would use eHealth to manage their RA if it were recommended by a doctor.	Patients’ habits and disease activity may also affect the usefulness of eHealth (e.g., patients in sustained remission might regard the use of eHealth for disease monitoring as futile)	
			eHealth device characteristics such as ease of use and security were mentioned by 31.8% and 20.9% of patients, respectively, to increase their adherence to eHealth.		
Seppen *et al***.** 2022 [50]	RCT (Netherlands)	To assess the safety and efficacy of patient-initiated care via a digital health application in patients with RA	Patient-initiated care supported by smartphone self-monitoring was noninferior to usual care in terms of the ΔDAS28-ESR and led to a 38% reduction in rheumatologist consultations in RA patients with stable low disease activity.	During the trial, 24 bug reports were made regarding 8 different bugs in the application.	A cost effectiveness evaluation of this intervention should be performed.
				Notifications did not work between July 2019 and February 2021 for most patients.	For patients needing extra time to become familiar with the app, an eHealth walk-in clinic could be organized.
Li *et al***.** 2023 [51]	RCT (Canada)	To assess the impact of a digital health application self-monitoring app, OPERAS and remote counselling PT service on patients with RA	Digital self-monitoring tools with remote counselling by a physiotherapist improved self-management ability in patients with RA.	Results may not be generalizable to people with RA who were at a lower level of activation and had different needs for self-management support.	Physiotherapists play a key role in supporting patient self-management regardless of geographic locations.
			The remote intervention improved rheumatoid arthritis disease activity, fatigue, depression and self-reported walking habit.		Further research on the cost-effectiveness of remote interventions will facilitate its integration in the care for patients with RA.
Watson *et al***.** 2024 [52]	Multicentre Mixed Methods Study (London, UK)	To evaluate a remote monitoring service using a Digital Pathway Coordinator in patients with RA	Service-level data showed high levels of patient engagement with the service. The patient survey showed patients felt the service was easy to use, had confidence in it and felt it improved access to care. Patient interview findings mirrored those of the survey.	Views from staff interviews were more mixed. Prioritization of staff needs was emphasized. Negative views of staff included fear of increased workload, loss of ‘control’ over patients and lack of ‘confidence’ in the service, and resistance to change.	Equal levels of patient and staff engagement are required for sustainability.
			Motivating factors included increased responsiveness and ease of contact with clinical teams.	Some patients felt the RAID prompts acted as an unwelcome reminder of their disease.	The potential value of integration with existing platforms, particularly the patient electronic health record, was emphasized.

HRQL: Health Related Quality of Life; JIA: Juvenile Idiopathic Arthritis; PEPPI: Patient-Physician Information Exchange Questionnaire; PT: Physiotherapy; RA: Rheumatoid Arthritis; RAID: Rheumatoid Arthritis Impact of Disease

**Table A5:** Showing studies involving Digital Diagnostics Decision Support Systems (DDSS)

Study ID	Design	Technology & Aim	Outcome	Challenges	Future Implications
Knitza *et al***.** 2022 [54]	Multicentre, open-label, crossover randomized controlled trial (Germany)	To evaluate patient experiences, acceptability and potential impact with a DDSS: Mobile AI–based symptom checker (Ada) and a web-based self-referral tool (Rheport)	Patients increasingly assess their symptoms independently online, however only a minority used dedicated symptom assessment websites or DDSS.	Although this study included three centres, the findings are limited to one country and patients referred to rheumatology services.	DSS could replace online search engines for patient symptom assessment, potentially saving time and increasing helpfulness.
			DDSS, such as Ada and Rheport are easy to use and well accepted among patients with musculoskeletal complaints. 44.3% and 65.8% of patients rated the respective symptom assessment as very helpful or helpful, using online search engines, Ada and Rheport, respectively	The study did not measure in how many cases patients needed help to use the DDSS. The usability rating of older patients of both DDSS was lower than in younger participants (p = 0.005).	From a physician perspective, the possibility to obtain a structured summary of the patient medical history to guide the appointment with importation into the electronic health record appeals time saving.
			Usability of both diagnostic decision support systems (DDSS) was “good” with a significantly higher mean score of Rheport (specific to rheumatology) compared to Ada (not rheumatology specific) (p < 0.0001).	Due to Ada’s variable questioning approach, determining new questions on all previously supplied basic health information, Ada bears the risk of leaving out important questions compared to a fixed-questionnaire approach (Rheport).	Picture incorporation might aid to specify definitions to reduce interpretation differences of symptoms
Knitza *et al***.** 2024 [55]	Prospective, multicentre, open-label, crossover randomized controlled trial (Germany)	To evaluate the diagnostic accuracy of a DDSS: Mobile AI–based symptom checker (Ada) and a web-based self-referral tool (Rheport) in patients with IRDS.	The diagnostic accuracies and agreement of both DDSSs for IRDs were not promising in this high-prevalence patient population.	DDSSs may lead to a misuse of scarce health care resources.	There is a need for stringent regulation and drastic improvements to ensure the safety and efficacy of DDSSs
			Physicians and the complex process of establishing a medical diagnosis cannot be replaced by an algorithm-based or AI-based DDSS.	The results of this study are not automatically transferable to other disciplines, languages, patient groups, and DDSS	The integration of large language models such as ChatGPT could significantly improve the DDSS performance

AI: Artificial Intelligence; IRDS: Inflammatory Rheumatic Diseases

**Table A6:** Showing Studies Involving a Cloud Platform

Study ID	Design	Technology & Aim	Outcome	Challenges	Future Implications
Richter *et al***.** 2021 [56]	Cross-sectional study (Germany)	An evaluation of the PICASO Cloud Platform on RA patients	Evaluations yielded predominantly positive feedback: the innovative design offering time-efficient data visualization, comprehension, and personalization was well accepted, and clinicians recommended further platform use for 93% of their patients.	Mainly technical reasons as “the platform or data were not accessible” and only in one case “lack of time” were given for non-use.	Effects on patients’ outcome, behaviour, and changes in the health care systems should be explored in larger sample populations by implementing ICT-based platforms enriched by upcoming Artificial Intelligence (AI) features where possible.
			86% of patients’ visits were managed using the clinician dashboard.	The dashboard approach may require adjustments once additional variables not previously existing are to be included.	Continuous use of such platforms like PICASO, with regular documentation of comprehensive measurements, can result in a big data scenario which facilitates additional developments of AI models to optimize multimorbidity research and management
			Platform use lead to improved disease and comorbidity management, with 70% of physicians reporting usefulness to assess patients’ diseases.	The platform was only tested with specific medical devices given out by the PICASO team.	
			At the end of the project, the majority of patients 93%, would recommend the platform to others, and about one-fifth (19), would like to continue using the platform even if it would become subject to charge.		
			Use of the platform assisted with patient empowerment, 48% set themselves new health-related goals, 92% stated easier patient-physician communications.		

ICT: Information and Communication Technology
